# Vitamin D status among apparently healthy individuals in the UAE: a systematic review

**DOI:** 10.3389/fnut.2025.1604819

**Published:** 2025-07-09

**Authors:** Maitha Abdulla Alshamsi, Wafeeqa Fatima, Maitha Tareq Al Teneiji, Suresh Kumar Srinivasamurthy

**Affiliations:** ^1^RAK College of Medical Sciences, RAK Medical and Health Sciences University, Ras Al Khaimah, United Arab Emirates; ^2^Department of Pharmacology, RAK College of Medical Sciences, RAK Medical and Health Sciences University, Ras Al Khaimah, United Arab Emirates

**Keywords:** systematic review, vitamin D levels, in UAE, vitamin D deficiency, healthy population

## Abstract

**Background/objectives:**

Vitamin D deficiency is a global health concern, particularly in regions with abundant sunlight, such as the UAE. This study aims to systematically review and meta-analyze available data on vitamin D levels in apparently healthy individuals in the UAE, categorizing findings by demographic factors, including age, gender, and ethnicity. The goal is to assess the extent of deficiency and identify potential contributing factors.

**Methods:**

A systematic review was conducted following PRISMA guidelines. PubMed and SCOPUS databases were searched for studies reporting serum vitamin D levels in healthy individuals in the UAE. Eligible studies included cross-sectional, retrospective, prospective, and comparative designs. Data were extracted and analyzed, with vitamin D levels categorized as deficient, insufficient, or normal. Study quality was assessed using a modified Newcastle–Ottawa scale for single-arm studies.

**Results:**

A total of 35 studies involving 28,260 participants were included. Reported vitamin D levels ranged from 5.2 ± 2.8 ng/mL to 42.5 ± 19.5 ng/mL. The pooled mean (SD) for adults above 18 years was 17.63 ng/mL (95% CI: 14.28 to 20.99) indicating widespread deficiency. Among participants, 65% were female, 34% were male, and 1% were infants. Severe deficiency was noted in infants and children, though limited studies focused on these groups.

**Conclusion:**

Despite high sunlight exposure, vitamin D deficiency is prevalent in the UAE, likely due to cultural clothing practices, limited outdoor activities, darker skin pigmentation, and dietary insufficiencies. Further research on vulnerable populations is needed. Variations in assay methods used across studies (e.g., RIA, ECLIA, LC–MS/MS) may have influenced reported vitamin D levels and contributed to heterogeneity in findings.

**Systematic review registration:**

https://www.crd.york.ac.uk/PROSPERO/view/CRD42024587972, Identifier, CRD42024587972.

## Introduction

Vitamin D deficiency is a widespread global issue in developed and developing countries, affecting approximately 1 billion people, with an additional 50% of the global population experiencing insufficiency (1). As a fat-soluble vitamin, it plays a crucial role in maintaining bone health, supporting immune function, and promoting overall well-being (2). Vitamin D, beyond its traditional role in bone health, acts as a secosteroid hormone regulating over 200 genes involved in immunity, inflammation, and cell function. Its deficiency is now associated with various conditions, including autoimmune, cardiovascular, metabolic, and neurodegenerative diseases, as well as certain cancers. The evidence also highlighted vitamin D role in mechanisms like NF-κB suppression, mitochondrial regulation, and gut microbiome modulation (3). Recent study suggests vitamin D supplements can reduce cancer-related mortality by 15% (4).

Vitamin D is synthesized in the skin when exposed to sunlight, specifically ultraviolet B (UVB) rays. When UVB light hits the skin, it converts a compound called 7-dehydrocholesterol, found in the skin, into vitamin D3 (cholecalciferol). This form of vitamin D is then transported to the liver, where it is converted into a substance called 25-hydroxyvitamin D (calcidiol). Finally, it travels to the kidneys, where it is converted into the active form of vitamin D, called calcitriol. This active form helps the body absorb calcium and phosphate, which are important for bone health and other bodily functions (5).

The prevalence of hypovitaminosis D (25(OH)D < 20 ng/mL) in the Middle East and North Africa varies across populations, ranging between 12 and 96% in children and adolescents, 54–90% in pregnant women, and 44–96% in adults (6). A study conducted in Abu Dhabi with 12,346 participants found that 72% of participants were vitamin D deficient (<20 ng/mL) and 10% were insufficient (20–30 ng/mL). This study also showed the high prevalence of vitamin D deficiency is consistent across both sexes, with 83.1% of males and 83.8% of females being deficient (7).

Despite the abundance of sunshine in the UAE, with an average of 10 h of sunlight per day, vitamin D deficiency remains prevalent. The severity of the situation is further highlighted by a large-scale study involving 7,924 patients in Dubai, which reported an overall mean serum 25(OH)D level of approximately 20 ng/mL, with 85.4% of the population being vitamin D deficient (8).

Though many studies have reported vitamin D levels from the UAE, there is a need for a systematic review of all these research studies to collate and analyze data on reported vitamin D levels. Hence this study was planned to report vitamin D levels in the apparently healthy population in the UAE as measured by liquid chromatography such as LC–MS–MS or by immunoassays, to find out the age group in which vitamin D deficiency is the most prevalent, and to identify the extent of vitamin D deficiency in various demographic groups within the UAE. The review also explores to evaluate methodological variations among included studies.

This review aims to support policymakers in the UAE by providing evidence-based insights into the burden and distribution of vitamin D deficiency, thereby informing public health interventions, screening programs, and national guidelines.

## Methodology

The study protocol was registered with Prospero (CRD42024587972). This systematic review complied with the Preferred Reporting Items for Systematic Reviews and Meta-Analyses (PRISMA) guidelines for reporting (9).

### Literature search

We searched PubMed and SCOPUS databases using the following search terms [(“vitamin d” OR cholecalciferol OR 1,25-dihydroxycholecalciferol) AND (uae OR “united arab emirates”)] for original articles on vitamin D serum levels in the apparently healthy UAE population (locals and non-locals) published from inception till July 1st, 2024.

**Figure 1:**
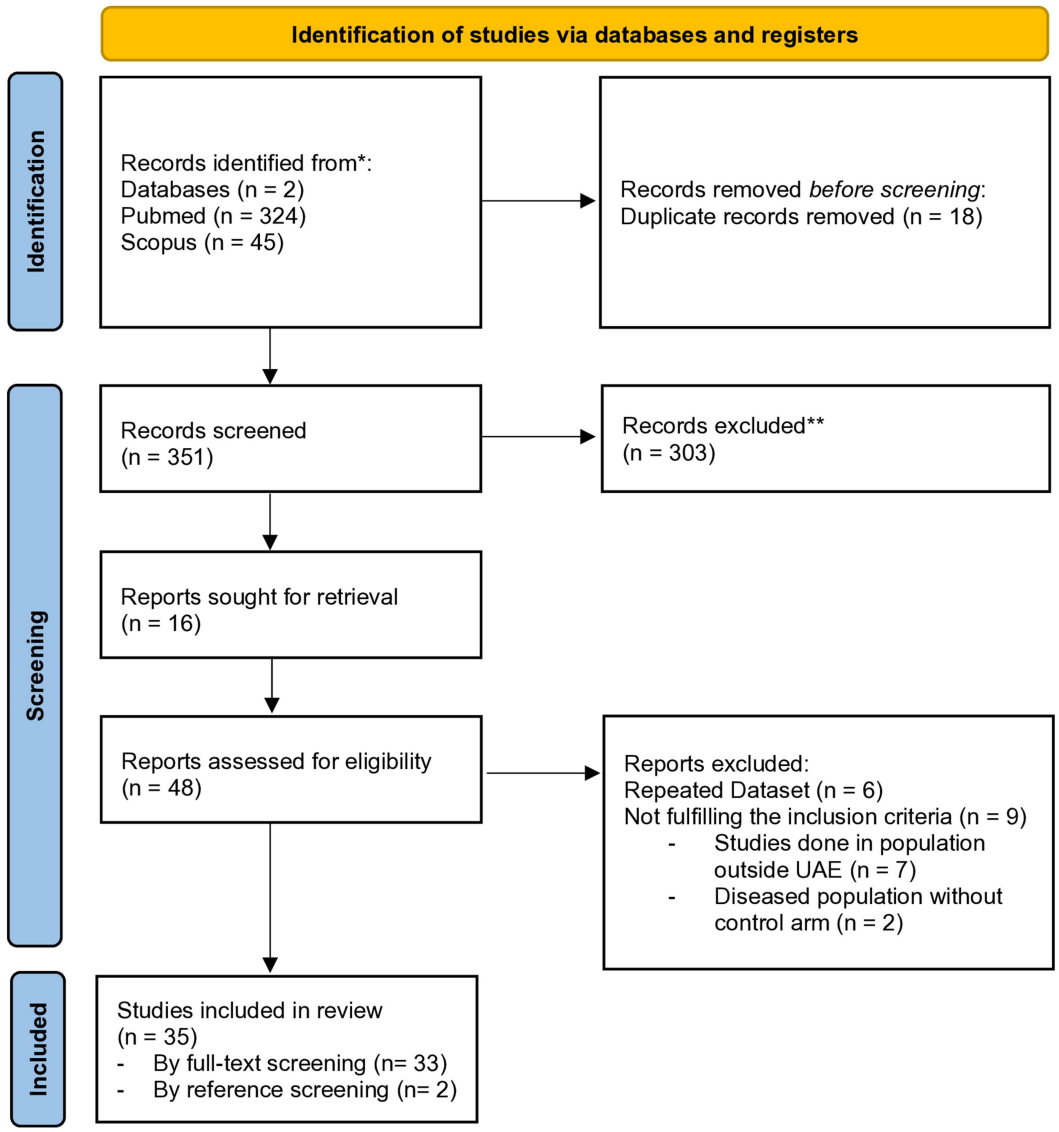
PRISMA flowchart describing the selection of studies for the systematic review. *We searched two electronic databases The number of records identified from each database is reported separately. **No automation tools were used for screening or exclusion; all records were assessed manually by reviewers.

We manually screened for the articles without using any software. We divided the procedure into 4 steps. In step 1, we screened using search terms. A total of 369 records were generated (324 articles from PubMed and 45 articles from SCOPUS), out of which 18 were excluded due to duplication, giving us 351 articles. Duplicate studies were identified by cross-checking article titles. In step 2 - we screened by the title and abstract, out of which 303 were excluded (276 articles from PubMed and 27 articles from SCOPUS) and 48 included (from PubMed only). In step 3 - we screened the 48 articles by full text, out of which 6 articles had repeated datasets and 9 articles did not fit our criteria. Hence these 15 articles were excluded, and the remaining 33 included. In step 4 - we screened the references of the included articles and found additional 2 articles, which fit our criteria. Hence a total number of 35 articles were included in this systematic review (Figure 1).

### Study selection

Studies were included in the present review if they met the following criteria: (1) outcome: articles reporting Vitamin D levels in the apparently healthy population (control population); (2) timeline: all articles published from the inception till July 1, 2024; (3) study participants: studies including all age groups and multiple ethnicities in the UAE (including locals and residents). (4) study designs: cross-sectional, retrospective, prospective, and comparative studies.

All studies conducted exclusively on patients with specific conditions, such as diabetes, hypertension, obesity, prematurity, infertility, and others, were excluded, Along with studies done on patients taking vitamin D supplements. Additionally, case reports, conference papers, *in vitro* studies, animal studies, and articles written in languages other than English were excluded.

### Data extraction

We organized the data from the included 35 articles into a master table made in an Excel sheet. All studies were independently screened and evaluated for selection by 3 authors (WF, MT, MA) initially. Once the data was collected it was cross-checked twice by the authors (WF, MT, MA). And it was finally reviewed for the third time by one of the authors (SK). Each study was evaluated using a data extraction form. For each study, we assessed a wide range of variables including Author Name, Year of Publication, Study designs, Number of arms (groups), Definition of controls, Study population age range, Mean age, Gender and ethnicity, Sample size, Sample per arm, Methods of assay of vitamin D, Quality assurance of lab methods, Sample (venous blood and saliva), Reported season, Reference level, Reported value, Converted value (to ng/mL), Standard deviation (SD), Percentages of vitamin D deficiency (VD-D), Insufficiency (VD-I), and Normal (VD-N), Reported values in subgroups, Converted values in subgroup (to ng/mL), Conclusion, Additional data, Ethics committee, Informed consent, Sample size calculations, and Statistics.

The quality analysis was done using the modified Newcastle–Ottawa scale for single-arm studies (10). The scoring was based on five specific parameters: definition of control, reported vitamin D, quality assurance of laboratory methods used for vitamin D measurement, ethics committee approval, and sample size calculation. Each study was scored based on these five parameters, with 1 point assigned for each criterion met, resulting in a total score out of 5.

Articles were systematically categorized into three demographic groups: mothers and infants, under 18 years of age, and above 18 years of age. The random-effects model was used for meta-analysis for the studies reporting subjects above 18 years category using R package version 4.4.2. Below 18 years and mothers and infants were not included in the meta-analysis due to sparsity in the number of studies. The conversion of data with respect to units and from interquartile ranges was done from a metaanalysis accelerator (11).

## Results

The studies included in the systematic review consisted of 35 studies involving 28,260 subjects. The studies reporting among subjects above 18 years (*n* = 26,129; Table 1), 1–18 years (*n* = 1,073; Table 2), and mothers with infants (*n* = 1,058; Table 3) were segregated and reported separately.

**Table 1:** Summary of studies reporting vitamin D levels in adults aged 18 years and above.

Author name	Study design	Number of arms (groups)	Study population age range	Study population age (yr.) mean (SD)	Study population gender	Study population ethnicity	Sample size	Sample per arm	Reported value	Converted value (ng/mL) mean (SD)
Dawodu et al. (26)	Pilot Study	3 (Emirati, Non-Gulf Arabs, and Europeans)	19–44 years	Emirati: 23.6 (18·5–28·7); Non-Gulf Arabs: 25·8 (19·6–32·0); Europeans: 32·7 (28·5–36·9)	Females	Emirati, non-Gulf Arabs, and Europeans	*N* = 75	Emirati (*n* = 33); Non-Gulf Arabs (*n* = 25); European (*n* = 17)	Not mentioned	Not applicable
Saadi et al. (32)	Not mentioned	2 (Premenopausal and Postmenopausal)	20–75 years	Median (range): 39 (20–72)	Females	Emirati	*N* = 56	Premenopausal (*n* = 38); postmenopausal (*n* = 18)	Premenopausal = 25OHD * ≥ 30 nmol/L: 39.3 ± 8.0 nmol/L; * < 30 nmol/L: 21.1 ± 5.2 nmol/L Postmenopausal: not mentioned	Premenopausal = 25OHD* ≥ 12 ng/mL: 15.7 ± 3.2 ng/mL; * < 12 ng/mL: 8.5 ± 2.1 ng/mL Postmenopausal: not mentioned
Saadi et al. (33)	Not mentioned	2 (Premenopausal and Postmenopausal)	20–85 years	Premenopausal: 37.5 ± 9.5; Postmenopausal: 58.3 ± 8.9	Females	Emirati	*N* = 259	Premenopausal women (*n* = 175); Postmenopausal (*n* = 84)	25.3 ± 10.8 nmoL/L	10.1 (4.3) ng/mL
Dawodu et al. (27)	Pilot study	1	20–30 years	Median (range): 24 (23–28)	Females	Arabs	*N* = 8	Nil	Median (range) = 17.6 (3.8–23.8) nmol/L	6.3 (2.78) ng/mL
Al Anouti et al. (17)	Cross sectional study	1	Not mentioned	Males: 21.0 ± 4.6; Females: 20.8 ± 4.0	Females (*n* = 208); Males (*n* = 70)	Emirati	*N* = 278	Nil	23.1 ± 15.5 nmol/L	9.3 (6.2) ng/mL
Al-Anouti et al. (20)	Not mentioned	1	Not mentioned	Median 43 (35–49)	Males (88%); Females (12%)	Emirati, Non-Gulf Arabs, Europeans, Canadians, and South Asians	*N* = 141	Nil	Median 22 (17 to 31) nmol/L	9.33 (4.19) ng/mL
Yammine et al. (8)	Cross-sectional retrospective study	1	Not mentioned	Mean age 37	Males (*n* = 2,418); Females (*n* = 5,506)	Locals and Non-locals	*N* = 7,924	Nil	19.9 (11.3) ng/mL	Conversion not needed
Anouti et al. (24)	Not mentioned	2 (VTD sufficient and VTD insufficient)	Not mentioned	VTD insufficient: 19.72 ± 0.19; VTD sufficient: 20.15 ± 0.55	Males (*n* = 52); Females (*n* = 111)	Emirati	*N* = 163	VTD insufficient (*n* = 136); VTD sufficient (*n* = 27)	13.52 ± 6.86 ng/mL	Conversion not needed
Bani-Issa et al. (25)	Cross-sectional study	1	18–64 years	Not mentioned	Females (*n* = 136); Males (*n* = 80)	Emirati and non-Emirati	*N* = 216	Nil	Not mentioned	Not applicable
Inman et al. (30)	Pilot study	1	≥ 18 years	29.08 ± 8.45	Males (*n* = 200); Females (*n* = 104)	Emirati	*N* = 331	Nil	21.36 (10.28) ng/mL	Conversion not needed
Hasan et al. (29)	Cross-sectional study	1	Not mentioned	Median age 21 (9) years	Males (*n* = 50); Females (*n* = 148)	Arab	*N* = 198	Nil	25.5 (18.2) (nmol/L)	10.2 (7.28)ng/mL
Thomas et al. (36)	Convenience sample	1	Not mentioned	20.83 (3.98) years	Females	Emirati	*N* = 114	Nil	23.66 (SD = 12.31) nmol/L	9.5 (4.9)ng/mL
Nimri et al. (31)	Cross-sectional study	1	18–26 years	20.19 (1.82)	Females	Not mentioned	N = 480	Nil	21.67 ± 9.5 ng/mL	Conversion not needed
Abdulle et al. (16)	Case–control study	2 (T2D and controls)	≥ 18 years	Controls - 50.7 ± 15.4	Control-Females (72.6%); Males (27.4%)	Emirati	*N* = 431	Control (*n* = 215); T2D Cases (*n* = 216)	Controls—29.3 ± 13.4 ng/ml	Conversion not needed
Al Zarooni et al. (7)	Cross-sectional retrospective observational study	1	18–106 years	Mean of 38.5 years	Male (*n* = 4,561); Female (*n* = 7,785)	Emirati	*N* = 12,346	Nil	Not mentioned	Not applicable
Sharif-Askari et al. (35)	Not mentioned	2 (insulin sensitive and insulin resistant)	18 to 80 years	Insulin sensitive- 38 (12)	Insulin sensitive- Males (*n* = 862); Females (*n* = 357)	Arabs, Asian, others	*N* = 4,114	Insulin sensitive (*n* = 1,354); insulin resistant (*n* = 2,760)	Median (IQR) of Insulin sensitive: 31.20 (21.4) ng/mL	Conversion not needed
Al-Amad et al. (19)	Case–control study	1	> 18 years	Mean of 31 years	Males (*n* = 32); Females (*n* = 20)	Not mentioned	*N* = 52	Nil	51.5 (26.9) nmol/l	20.6 (10.8) ng/mL
Saeed et al. (34)	Cross-sectional and prospective	1	18–26 years	19.9 ± 1.6	Males (*n* = 98); Females (*n* = 189)	Emirati, Arab non-Emirati, and Non-Arab	*N* = 287	Nil	15.8 (19.5 ± 11.6) ng/ml	Conversion not needed
Gariballa et al. (28)	Randomized controlled trial	1	≥ 18 years	41 ± 12	Males (*n* = 73); Females (*n* = 204)	Emirati and Arab	*N* = 277	Nil	23.7 (11)ng/ml	Conversion not needed
Al Zarooni et al. (18)	Cross-sectional retrospective observational study	1	18–70 years	38.9 (13.2) years	Females (61.5%); Males (38%)	Emirati	*N* = 392	Nil	Mean vitamin D levels of physically active: (38.2 nmol/L), Physically inactive: (31.7 nmol/L). Depending on the nature of work: sedentary work (29.6 nmol/L), physically demanding work (37.1 nmol/L).	Mean vitamin D levels of physically active: (15.3 ng/mL), Physically inactive: (12.7 ng/mL). Depending on the nature of work: sedentary work (11.9 ng/mL), physically demanding work (14.9 ng/mL).
Anouti et al. (23)	Cross-sectional study	1	≥ 18 years	35 (10)	Females	Philippines, Arab, and South Asian	*N* = 553 *there is discrepancy	Nil	20 ± 11 ng/mL	Conversion not needed
AlAnouti et al. (21)	Not mentioned	1	≥ 18 years	Median (IQR) = 30 (23, 38)	Males (*n* = 281); Females (*n* = 118)	Emirati	*N* = 399	Nil	Median (IQR) = 19.5 (15.5, 25.6) ng/mL	20.2 (7.51) ng/mL
Alzohily et al. (22)	Randomized, double-blind, placebo-controlled trial	3 baseline (vitamin D deficient obese), follow-up (supplemented obese), and healthy volunteers	Age range of healthy males (18–29), and females (18–65)	Not mentioned	Healthy volunteers- Males (*n* = 8); Females (*n* = 167)	Emirati citizens and Middle Eastern expatriates	*N* = 452	Baseline and Follow-up (*n* = 277); Healthy volunteers (*n* = 175)	Healthy volunteers 25OH vitamin D3: 12.56 ± 3.84 ng/ml	Conversion not needed

VTD, Vitamin D; T2D, Type 2 diabetes.

**Table 2:** Summary of studies reporting vitamin D levels in individuals below 18 years of age.

Author name	Study design	Number of arms(groups)	Study population age range	Study population mean SD (Age)	Study population gender	Study population ethnicity	Sample size	Sample per arm	Reported value	Converted value (ng/mL)	SD
Rajah et al. (15)	Prospective study	4 (0–0.9 y, 1–1.9 y, 2–7.9 y, 8–14 y)	< 14 y	5.32 (3.76)	Males (*n* = 88); Females (*n* = 81)	Not mentioned	*N* = 169	0–0.9 y (*n* = 16); 1–1.9 y (*n* = 26); 2–7.9 y (*n* = 79); 8–14 (*n* = 48)	53.6 (33.4) nmol/L	21.5 (13.4) ng/mL	13.4ng/mL
Muhairi et al. (13)	Cross-sectional study	1	12 to 18 years	Mean age (SD) was 16 years for females (0.6) as well as for males (0.9)	Male (*n* = 150); Female (*n* = 165)	Emirati, and others (Arab, and Gulf countries).	*N* = 315	Nil	Mean serum 25 (OH) D concentrations in Emirati females: 20.8 ng/mL (95% CI 18.8–22.8), Emirati males: (mean 25.2 ng/mL; 95% CI 23.7–26.8), non- Emirati females: 21.9 ng/mL (95% CI; 19.8–23.9), non- Emirati males (mean 28.3; 95% CI 24.6–31.9).	Conversion not needed	Not mentioned
Narchi et al. (14)	Cross-sectional Analytical study	1	11–18 years	15.2 (2.0)	Females	Emirati	*N* = 293	Nil	21.5 (10.0) nmol/L.	8.6 (4) ng/mL	4 ng/mL
Majeed et al. (12)	Case control study	2 (T1D cases and NG controls)	Not mentioned	NG controls—11.9 (10.1, 14.6)	NG controls—Female (*n* = 173); Male (*n* = 123)	Emirati	*N* = 444	T1D cases (*n* = 148); NG controls (*n* = 296)	NG controls -Median (IQR): 34.9 (23.6, 50.5) (nmol/L)	NG controls - median (IQR): 14 (9.5, 20.2) ng/mL	Not mentioned

T1D, Type 1 Diabetes; NG, normoglycemic.

**Table 3:** Summary of studies reporting vitamin D levels among mothers and their infants.

Author name	Study design	Number of arms(groups)	Study population age range	Study population mean SD (Age)	Study population gender	Study population ethnicity	Sample size	Sample per arm	Reported value	Converted value (ng/mL)	SD
Dawodu et al. (38)	Not mentioned	2 (mothers and (infants and young children))	Infants and young children: 5–35 months	Infants and young children mean: 15.4 months	Infants and young children: (Male / Female): 31/20	Infants and children: Emirati (*n* = 26); non-Gulf Arabs (*n* = 25)	*N* = 101	Infants and young children (*n* = 50)	Infants and children: 49.8 (30.8) nmol/L	Infants and children: 20 (12.3)ng/mL	Infants and children: 12.3ng/mL
			Mother: not mentioned	Mothers: not mentioned	Mothers: females	Mothers: not mentioned		Mothers (*n* = 51)	Mothers: 28.4 (14.0) nmol/L	Mothers: 11.4 (5.6)ng/mL	Mothers: 5.6 ng/mL
Dawodu et al. (39)	Not mentioned	2 (mothers and infants)	Infants: 4 to 16 weeks	Median age of infants: 6 weeks	Infants: not mentioned	Infants: Arab and South Asian	*N* = 168	Infants (*n* = 78)	Infant median (quartiles): 4.6 (2.5, 7.9)ng/mL	Conversion not needed	Not mentioned
			Mothers not mentioned	Mean age of the mothers was 27.1 ± 6.0 years.	Mothers: female	Mothers not mentioned		Mothers (*n* = 90)	Mothers median (quartiles): 8.7 (5.9, 13.6)ng/mL	Conversion not needed	Not mentioned
Saadi, et al. (44)	Not mentioned	3 (breastfeeding women receiving daily regimen, breastfeeding women receiving monthly regimen, and infants receiving daily regimen)	Not mentioned	Infants of mothers*receiving daily regimen (*N* = 45) 18.9 ± 22.5 *receiving monthly regimen (*N* = 47) 22.4 ± 25.6	Infants: not mentioned	Infants not mentioned	*N* = 182	Infants (*n* = 92)	Infants of mothers *receiving daily regimen (*N* = 45) 13.1 ± 7.1 nmol/L *receiving monthly regimen (*N* = 47) 15.0 ± 10.9 nmol/L	Infants of mothers *receiving Daily regimen (*N* = 45) 5.2 ± 2.8 ng/mL *receiving Monthly regimen (*N* = 47) 6 ± 4.4 ng/mL	Infants of mothers*receiving Daily regimen (*N* = 45) SD: 2.8 ng/mL *receiving Monthly regimen (*N* = 47) SD: 4.4 ng/mL
				Mothers *receiving daily regimen: 29.2 ± 5.5; *receiving monthly regimen 29.9 ± 6.7	Mothers: Female	Mothers: Arabs (*n* = 76) and South Asians (*n* = 14)		Healthy breastfeeding mothers (*n* = 90) *Receiving daily regimen (*N* = 45) *Receiving monthly regimen (*N* = 45)	Mothers *receiving daily regimen: 27.3 ± 10.4 nmol/L; * receiving monthly regimen 23.2 ± 10.7 nmol/L	Mothers *receiving daily regimen: 10.9 ± 4.2 ng/mL *receiving monthly regimen 9.3 ± 4.3 ng/mL	Mothers *receiving Daily regimen (*N* = 45) SD: 4.2 ng/mL *receiving monthly regimen (*N* = 45) SD: 4.3 ng/mL
Amirlak et al. (37)	Convenience sample, Pilot study	2 (mothers and infants)	Not mentioned	Infants: not mentioned	Infants: not mentioned	Infants: not mentioned	*N* = 168	Infants (*n* = 84)	Infants cord blood median (IQR): 9.9 (7.6, 17.4) nmol/L	Infants cord blood median (IQR): 4 (3, 7) ng/mL	Not mentioned
				Median (IQR) age of the women was 27 (23; 31)	Mothers: Females	Mothers: Arab (*n* = 68), and South-East Asian (*n* = 16) (Indian and Pakistani)		Mothers (*n* = 84)	Maternal median (IQR): 18.5 (11.0, 25.4) nmol/L	Maternal median (IQR): 7.4 (4.4, 10.2) ng/mL	Not mentioned
Narchi, et al. (43)	Prospective longitudinal cohort study	1	18–40 years	Median age: 27 years	Females	Egyptian (*n* = 20), Palestinian (*n* = 16), Joardanian (*n* = 13) Sudanese (*n* = 8), others (*n* = 18)	*N* = 75	Nil	17.3 (10.5) ng/mL	Conversion not needed	10.5 ng/mL
Narchi, et al. (42)	Prospective, longitudinal cohort study	2 (mothers and infants)	Not mentioned	Infant median (range) * at birth: 3 days (2–7); * After 6 months: 4 weeks (12–32)	Infants: Males (*n* = 16), Females (*n* = 11)	Infants: not mentioned	*N* = 50	Infants (*n* = 27)	Infants mean (SD) *At birth: 44.7 (29.7)nmol/L; *After 6 months: 106 (48.7)nmol/L	Infants mean (SD) *At birth: 17.9 (11.9)ng/mL; *After 6 months: 42.5 (19.5)ng/mL	Infants (SD) *At birth: (11.9)ng/mL; *After 6 months: (19.5)ng/mL
				Mothers: not mentioned	Mothers: Females	Mothers: Middle Eastern and Asian origin		Mothers (*n* = 23)	Mothers mean (SD): 35.5 (24.7)nmol/L	Mothers mean (SD): 14.2 (9.9) ng/mL	Mothers SD: 9.9 ng/mL
Dawodu and Nath et al. (40)	Not mentioned	1	Not mentioned	Mean (SD): 27.0 (5.5) years	Females	Arab	*N* = 28	Nil	Maternal Median (IQR): 17.0 (12.0, 21.5) nmol/L	6.8 (4.8, 8.6) ng/mL	Not mentioned
Jutell, et al. (41)	Prospective observational study	3 (Group I: 25(OH)D < 25 nmol/L, Group II: 25(OH)D 25 nmol/L to < 50 nmol/L, Group III: 25(OH)D > 50 nmol/L)	Not mentioned	Group I: 29.8 ± 6.25,	Females	Middle east, Africa, Southeast Asia	*N* = 286	Group I (*n* = 64)	Group I: 17.5 ± 4.9 nmol/L	Group I: 7 ± 1.96 ng/mL	1.96 ng/mL
				Group II: 30.2 ± 5.24				Group II (*n* = 72)	Group II: 37.3 ± 7.55 nmol/L	Group II: 14.92 ± 3.0 ng/mL	3 ng/mL
				Group III: 30.9 ± 6.22				Group III (*n* = 150)	Group III: 81.7 ± 23.73 nmol/L	Group III: 32.68 ± 9.5 ng/mL	9.5 ng/mL

The Vitamin D levels reported across all 35 included studies ranged from 5.2 ± 2.8 ng/mL to 42.5 ± 19.5 ng/mL. Among children under 18 years of age, the lowest reported level was 8.6 ± 4 ng/mL, while the highest was 21.5 ± 13.4 ng/mL (12–15). For individuals over 18 years, the lowest level reported was 6.3 ± 2.78 ng/mL, and the highest was 31.20 ± 21.4 ng/mL (7, 8, 16–36).

Out of the total 28,260 subjects, 18,451 were female, 9,478 were male and 331 were infants whose gender was not specified. Male participants were 65%, females 34, and 1% infants.

Among 35 studies, we included 8 studies about moms and infants, however only 5 out of 8 studies met our inclusion criteria for infants (37–44). In addition, 8 studies included only female participants. The remaining 19 studies included both male and female participants. In this present systematic review, 11 studies were done on Emirati participants, among which 9 studies were conducted in above 18, 2 studies in under 18, and none in the mom and infants group.

The age range of participants widely varied across the included articles. Most of the studies focused on adults aged 18 years and above with some studies reporting age ranges. A few studies included broader age ranges such as 18–106 years, 20–85 years, and 20–75 years. There are studies specifying subgroups for example the participants within the fertile age range of 19–44 years (Supplementary Table 1).

**Figure 2:**
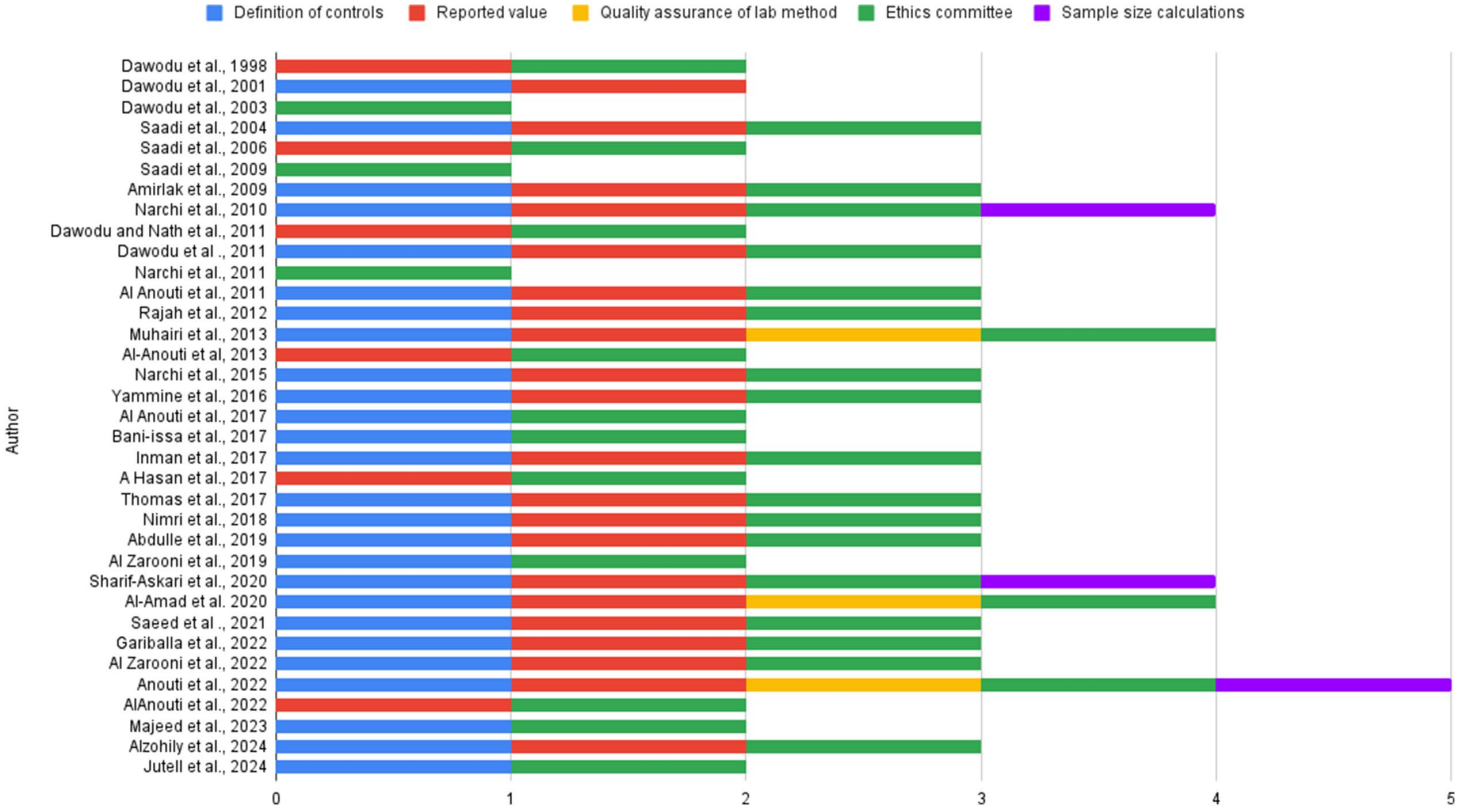
Quality assessment using the modified version of the Newcastle–Ottawa scale.

The average quality analysis score of studies as done by the modified Newcastle Ottawa scale is 2.65 (Figure 2). The mean score of quality analysis calculated for each group is as follows, 2.82 (above 18), 2.0 (mom and infants), and 3.0 (under 18; Supplementary Table 2).

**Figure 3:**
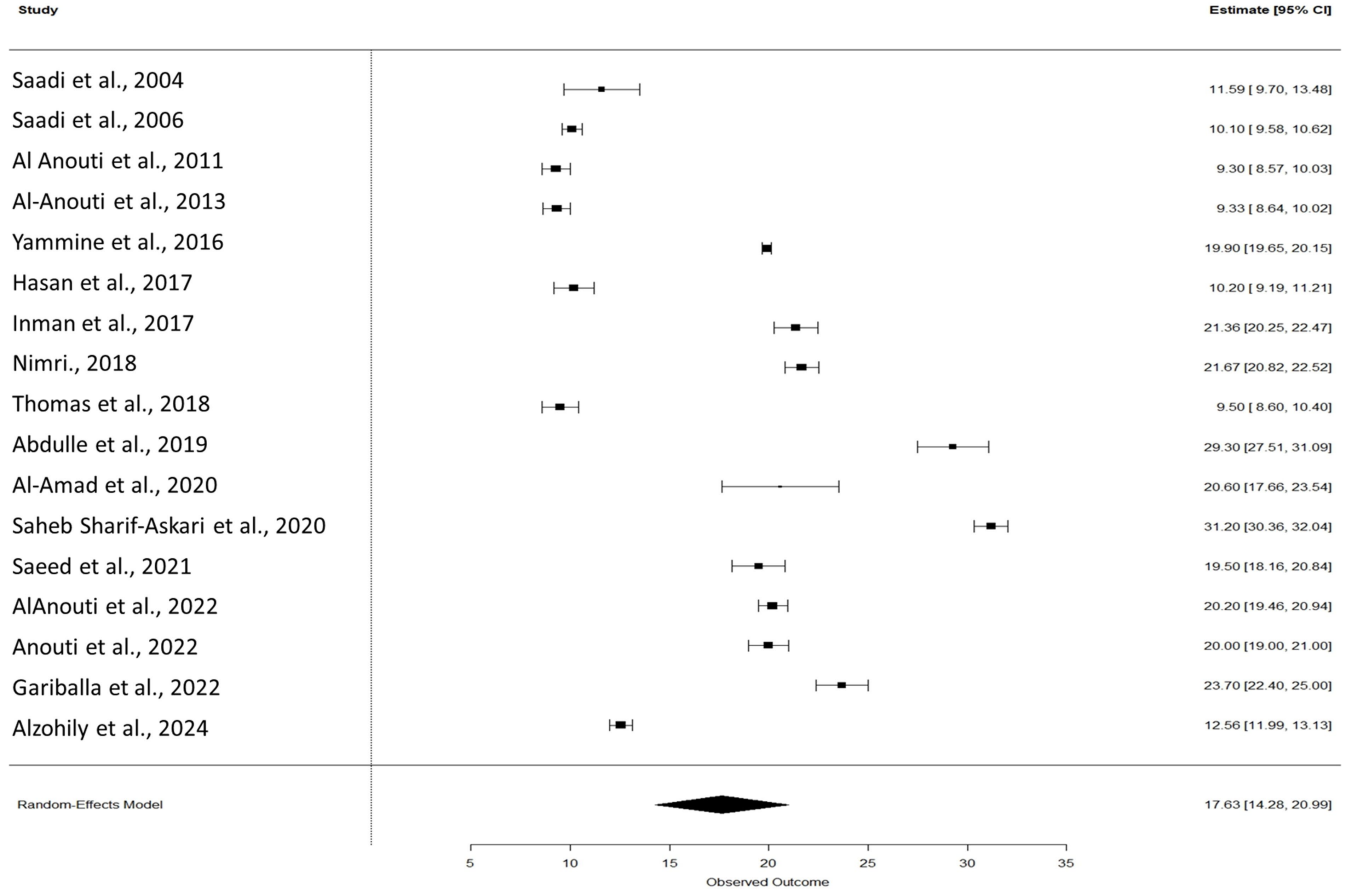
Forest plot of pooled vitamin D levels among studies. The model was estimated using the random effects model using R package version 4.4.2.

A meta-analysis of 17 studies was conducted using a random-effects model to estimate the pooled mean and explore sources of heterogeneity. The overall pooled mean was 17.63 (95% CI: 14.28 to 20.99), with very high heterogeneity observed (I^2^ = 99.71%, τ^2^ = 49.42, *p* < 0.0001; Figure 3). Metaregression moderator analysis indicated that the measurement method was a significant source of variation (QM = 14.84, *p* = 0.0381), with studies using combined methods, HPLC, and RIA reporting significantly lower mean values compared to the reference method. In contrast, sample size (*p* = 0.4795) and study location (*p* = 0.4724) were not significant moderators when examined independently. The full mixed-effects model, which included sample size, location, and method, explained approximately 43.8% of the heterogeneity (QM = 21.29, *p* = 0.0114), with the measurement method remaining a significant predictor. Sample size and location showed marginal or borderline effects in the full model, suggesting that measurement technique was the primary contributor to the observed between-study variability.

In adults above 18 years of age included in the meta-analysis, 5 out of 17 studies stated vitamin D deficiency as levels below 20 ng/mL, however, one study mentioned <20 ng/mL as insufficiency (21–23, 28, 35). Six studies included participants who were exclusively Emirati (16, 17, 21, 32, 30, 36). Thirteen studies assessed the correlation between Body Mass Index (BMI) and vitamin D level (16, 17, 20–23, 28–31, 33–35), of which only 5 found a statistically significant correlation (16, 23, 31, 34, 35).

Out of the 23 studies assessed in the above 18 years category, 17 were included in the meta-analysis, while 6 were excluded due to data limitations. Al Zarooni et al. (7), Bani-Issa et al. (25), and Al Zarooni et al. (18), Dawodu et al. (26) were excluded as they only provided categorical data. Dawodu et al. (27) was excluded due to a small sample size (*n* = 8), reducing its statistical reliability. We excluded Anouti et al. (24) from meta-analysis as it did not report results for the entire study population, and 13.52 ± 6.86 ng/mL was derived and not presented in the study (45).

In our systematic review, the most commonly used study design was the cross-sectional study, followed by prospective studies, randomized controlled trials, case–control studies, and pilot studies. Additionally, there were 9 articles, where the study design was not mentioned. All the studies were conducted with venous blood collected samples and none were with saliva.

Out of all the studies, Radioimmunoassay (RIA) was mostly used to measure the Vitamin D levels—9 times, followed by Electrochemiluminescence immunoassay, used 6 times. Other studies utilize chemiluminescence immunoassay, Immunoassay, Beckman Coulter analyzers, and a combination of methods for vitamin D assay. Whereas 4 studies did not mention the specific assay technique used. This variation in assay methods highlights the difference in measurement approaches across the included studies.

Among the 35 studies included in the systematic review, 21 utilized questionnaires as their primary assessment tool. Three studies assessed for depression; one used the Patient Health Questionnaire (PHQ-9), and 2 employed the Beck Depression Inventory (BDI). The remaining studies used a questionnaire focused on lifestyle factors, including average duration of sunlight exposure, dietary history, and dress style.

## Discussion

Our study revealed that the pooled mean (SD) obtained from studies reporting vitamin D levels is in the range of vitamin D deficiency. The studies included in the systematic review consisted of 35 studies (8–mom and infants, 4–under 18, 23–above 18; Tables 1–3) involving 28,260 subjects (*N* = 1,058—mom and infants, *N* = 1,073—under 18, *N* = 26,129—above 18).

The findings of meta-analysis highlight the substantial heterogeneity across studies, as indicated by the high I (2) value of 99.71%, suggesting that differences in study characteristics significantly influenced the reported outcomes (Figure 3). Among the examined moderators, the method of measurement emerged as a key source of heterogeneity. Studies employing combined methods, HPLC, and RIA consistently reported lower mean values compared to the reference method, underscoring the impact of analytical techniques on study results. In contrast, neither sample size nor study location significantly accounted for the observed variability when considered independently. However, when included in a mixed-effects model, the combination of moderators explained nearly 44% of the total heterogeneity, with the measurement method remaining a significant predictor. These results emphasize the need for standardization in measurement approaches across studies to improve comparability and reduce methodological bias in future research.

Overall our included studies from 1998 to 2024 have reported that vitamin D deficiency is significant in our population. This is in concurrence with another systematic review done in India (46). However, there is another systematic review of vitamin D status in populations worldwide which showed vitamin D levels in the normal range in certain countries (2).

The total studies included in the under 18 category are 4 with total subjects of 1,073. This also indicates that the data reporting on vitamin D levels among children and adolescents are sparse compared to adults.

In the same line, only 8 studies have reported vitamin D levels among mothers and infants. The vitamin D levels among mothers ranged from 7 ± 1.96 ng/mL to 32.68 ± 9.5 ng/mL (41) and among infants the range was from 5.2 ± 2.8 ng/mL to 42.5 ± 19.5 ng/mL (39, 42). The recommended normal vitamin D level for pregnant women and infants, as stated by the World Health Organization (WHO), is above 50 nmol/L (20 ng/mL) (47, 48).

Vitamin D deficiency is highly prevalent among both pregnant women and adolescents, but the priority and approach to intervention should differ: pregnant women require more urgent and higher-dose supplementation because deficiency is linked to serious maternal and neonatal complications such as preeclampsia, gestational diabetes, preterm birth, and low birth weight. Thus, interventions for pregnant women should be prioritized and integrated into prenatal care with routine monitoring (49, 50).

While adolescents though also at risk for deficiency-related bone and growth problems generally benefit from intermediate-dose supplementation and targeted screening mainly if risk factors are present. For adolescents, a population-level approach with supplementation and lifestyle advice is usually sufficient unless additional risks are identified (49, 51).

UAE is known for its desert climate and abundant sunshine throughout the year. Despite this, vitamin D deficiency remains a significant health problem. Factors contributing to this paradox include cultural clothing practices that decrease skin surface area exposed to sunlight, extremely hot weather that discourages outdoor activities, dark skin tones that reduce vitamin D synthesis, and insufficient intake of vitamin D-rich foods (18).

Out of the 35 studies reviewed, only three explicitly referenced the seasons during which data were collected. These included the study by Fatme Al Anouti et al. (17), conducted during both winter and summer; the study by Justin Thomas et al. (36), conducted in the fall; and the study by Adekunle Dawodu et al. (39), conducted in the summer. In contrast, 22 studies reported only a range of months without specifying the corresponding seasons, while the remaining studies did not mention the timing of data collection at all.

One study has reported seasonal variation in vitamin D levels between summer and winter. During the summer, vitamin D levels were lower, with females having an average of 8.4 ± 6 ng/mL and males 10.9 ± 6.3 ng/mL. In contrast, winter levels were higher, with females averaging 12.5 ± 4.9 ng/mL. The difference between summer and winter was statistically significant, suggesting that seasonal variations play a major role in vitamin D status. This variation is likely due to factors such as sunlight exposure, as intense summer heat may discourage outdoor activity, while cooler winter weather encourages more time outside, leading to greater vitamin D production (17).

A study reported that participants engaging in daily physical activity had the highest mean vitamin D levels (15.28 ng/mL), while those with minimal activity exhibited the lowest levels (12.68 ng/mL). Work nature also influenced vitamin D status, with individuals spending most of their time sitting at work showing the lowest mean levels (11.84 ng/mL). Whereas, participants whose jobs required physical exertion had higher mean levels (14.84 ng/mL) (18).

Similarly, cultural practices particularly clothing styles that limit skin exposure are major contributors. One study reported that 51% of mothers and 22% of children had serum 25-hydroxyvitamin D (25-OHD) levels below 10 ng/mL. Limited sun exposure (mean 38 min/day) and heavy clothing, with 95% of children exposing only their face and hands outdoors, were identified as key risk factors (38). Nimri et al. showed that the prevalence of low vitamin D levels varies significantly between individuals wearing hijabs and those adopting a Western dress style. Among individuals wearing hijab, 37.5% were found to have low vitamin D levels. In contrast, those following a Western dress style had a lower prevalence, with only 16.7% reporting low vitamin D levels (31). Another study highlighted that Emirati women of childbearing age had a mean serum 25-OHD level of 8.6 ng/mL, far below the 64.3 ng/mL observed in Europeans living in the UAE. This discrepancy correlated strongly with clothing styles that covered most of the body, reducing effective UVB exposure (26).

The factors for prevalent vitamin D deficiency are well-reviewed in multiple studies. Among them, the causes for the UAE context are limited sunlight exposure, urban areas, or those following indoor lifestyles (20). Cultural or religious practices requiring full-body coverings and darker skin pigmentation, which reduces the skin’s ability to synthesize vitamin D, also contribute significantly (26, 31, 38). Seasonal variations, particularly in summer, further exacerbate the vitamin D deficiency (17). Obesity, aging, and medications that interfere with vitamin D metabolism also increase vulnerability, as do exclusive breastfeeding without supplementation for infants (21, 44).

## Strengths

This systematic review, conducted in accordance with the 2020 PRISMA guidelines, is the first to report on vitamin D levels in the UAE. Data were collected from inception to date using two large databases. Articles were systematically categorized into three demographic groups: mothers and infants, under 18 years of age, and above 18 years of age. We accounted for variations in reference levels used to categorize deficiency, insufficiency, and normal vitamin D status across all articles, which offer a clear comparison and comprehensive interpretation of vitamin D status across different groups. Also, we assessed all our articles based on the modified version of the Newcastle–Ottawa scale, for quality assessment, scoring them between 0 to 5 (Figure 2).

## Limitations

We did not segregate the data based on the seasons, which could have influenced Vitamin D levels due to variations in sunlight exposure across the year. Individuals in the UAE may have greater exposure to sunlight during winter months which leads to higher vitamin D levels compared to the summer months. The study population in the UAE is heterogeneous and multi-ethnic, which may introduce variability that was not fully accounted for in the analysis.

One more significant limitation of this systematic review is the lack of information regarding the duration of stay of migrant participants in the UAE. This missing data could influence the interpretation of findings, as the length of stay may affect participants’ vitamin D values.

The scope of our research was limited, as key databases such as Embase, Web of Science, and Google Scholar were not included. We recommend that future systematic reviews incorporate these databases to enhance the comprehensiveness of the search.

### Future direction

Future research should explore vitamin D levels in the Emirati population to gain a deeper understanding of local trends, taking into account unique factors such as genetics, dietary habits, and cultural practices.

Additionally, studies examining seasonal variations in vitamin D levels considering the differences in sunlight exposure between summer and winter months, would provide valuable insights into the impact of seasonal changes on health. Conducting a longitudinal study to track the changes in vitamin D over time.

Moreover, studies with larger sample sizes should be prioritized to enhance the reliability and generalizability of findings. A more extensive sample would allow for greater statistical power and more robust subgroup analyses, enabling a better understanding of variations in vitamin D levels across diverse populations.

While cross-sectional and retrospective studies are effective for estimating prevalence, they limit causal inference. We recommend that future studies in the UAE employ long-term longitudinal or interventional designs to assess causality between lifestyle factors and vitamin D status. Further, future studies should underscore the need for targeted public health strategies, which should be explored in dedicated intervention studies.

In addition, there is a need to conduct systematic reviews specifically focused on the Emirati population. In our current systematic review, only 11 articles included exclusively Emirati participants. Targeting this population in future studies would provide more representative data and contribute to a better understanding of vitamin D status in the UAE.

## Conclusion

Vitamin D deficiency is a significant public health concern in the UAE, even among the apparently healthy population. This systematic review reveals that vitamin D deficiency is prevalent across various demographic groups, including different age ranges including mothers and infants, children (below 18 years) and adults (above 18 years), genders, and socio-economic states. The Vitamin D levels reported across all 35 included studies ranged from 5.2 ± 2.8 ng/mL to 42.5 ± 19.5 ng/mL. The pooled mean (SD) of vitamin D for adults above 18 years was 17.63 (CI 14.28 to 20.99). According to our analysis of the articles included in this systematic review, we found that infants had the lowest vitamin D levels.

Furthermore, differences in assay techniques across studies—such as radioimmunoassay (RIA), electrochemiluminescence immunoassay (ECLIA), and LC–MS/MS—can significantly impact measured vitamin D values and should be carefully considered when interpreting results or formulating public health guidelines.

## Data Availability

The original contributions presented in the study are included in the article/supplementary material, further inquiries can be directed to the corresponding author.
