# Supplementary material for: Vitamin D status among apparently healthy individuals in the UAE: a systematic review
Source: Front Nutr. 2025 Jul 9;12:1604819. doi: 10.3389/fnut.2025.1604819 (PMC12284947; doi:10.3389/fnut.2025.1604819)
Supplement: Supplementary file 1 [file Data_Sheet_1.pdf]

Table S1. The additional characteristics of the studies included in the systematic review

Author name	Method of assay of vitamin D	Sample (venous blood, saliva…)	Reported season	Reference level	Reported values in subgroups	Converted Value in Subgroups (ng/mL)	Additional data	Informed consent
Dawodu et al., 1998	HPLC	Blood	June and September 1994	Not mentioned	Group 1 (N=33) 8·6 (4·5–17·4); Group 2 (N=25) 12·6 (6·0–26·4); Group 3 (N=17) 64·3 (49·0–84·3) (ng/ml)*	Conversion not needed	The mean serum concentration of 25-OHD among female UAE nationals and other non-Gulf Arabs in this study is about one-fifth of the value for European women resident in the UAE.	Consent was obtained.
Dawodu et al., 2001	HPLC method	Blood	February through September	Not mentioned	The level of serum 25-OHD concentration in this 6-month-old infant was undetectable while that of his mother was 18.8 nmol/l	The level of serum 25-OHD concentration in this 6-month-old infant was undetectable while that of his mother was 7.5 ng/mL	The results suggest normal vitamin C status but a possible high prevalence of hypovitaminosis D in Arab children and their mothers in UAE.	Verbal parental consent had been obtained
Dawodu et al., 2003	High-performance liquid chromatography after extraction with acetonitrile and purification with a C-18 column	Not mentioned	summer of 1999 (April to October)	For mothers=11–68 (ng/mL); For infants= 10–52 (ng/mL)	Mothers: 8.7 (5.9, 13.6); Infants: 4.6 (2.5, 7.9) (ng/mL)	Conversion not needed	Serum 25-OHD concentrations in infants are very low compared with reports published from Western countries in the last two decades.	Parental consents obtained.
Saadi et al., 2004	Radioimmunoassay (DiaSorin Inc., Stillwater, Minnesota).	Not mentioned	Not mentioned	not mentioned	Premenopausal = 25OHD* ≥ 30 nmol/L (n = 9) (mean ± SD:39.3 ± 8.0	Premenopausal = 25OHD* ≥ 12 ng/mL (n = 9) (mean ± SD: 15.7. ± 3.2 ng/mL).; *< 12 nmol/L (n =	Forty-eight women (85.7%) had low calcaneal BMD estimated by QUS (T-score ≤ –1).	Informed consent

					nmol/L).; *< 30 nmol/L (n = 12) (mean ± SD: 21.1 ± 5.2 nmol/L) / post menopausal not mentioned	12) (mean ± SD: 8.5 ± 2.1 ng/mL) / post menopausal not mentioned		
Saadi et al., 2006	Radioimmunoassay (DiaSorin; Stillwater, Minnesota).	Blood	Jan 1, 2003 and June 30, 2005		Emirati *Premenopausal women (n = 175): 24.3 ± 10.4 *Postmenopausal women (n = 84): 27.3 ± 11.2 ; Non-Arab women 63.3 ± 26.2 (nmol/l)	Emirati *Premenopausal women (n = 175): 9.7 ± 4.2 *Postmenopausal women (n = 84): 10.9 ± 4.5; Non-Arab women 25.4 ± 10.5 (ng/mL)	Mean serum 25OHD was highest in April (29.2 ± 13.0 nmol/l), which marks the end of the short and cooler winter season, and lowest in August (18.2 ± 5.9 nmol/l).	Written informed consent
Saadi et al., 2009	Radioimmunoassay (DiaSorin; Stillwater, MN, USA)	Blood	September 2005–February 2006	Not mentioned	Baseline Serum 25(OH)D (nmol L−1): Total Mothers *receiving Daily regimen (N=45) 27.3 ± 10.4 *receiving Monthly regimen (N=45) 23.2 ±10.7; Total Infants *receiving Daily regimen (N=45) 13.1 ± 7.1 receiving Monthly regimen (N=47) 15.0 ± 10.9	Total mothers receiving Daily regimen (N=45) 10.9 ± 4.2 *receiving Monthly regimen (N=45) 9.3 ± 4.3; Total Infants *receiving Daily regimen (N=45) 5.2 ± 2.8 receiving Monthly regimen (N=47) 6 ± 4.4 ng/ml	There were no significant differences noted in the baseline characteristics between mothers (and infants) that completed the study and those who dropped out	Informed consent
Amirlak et al., 2009	Highperformance liquid	Venous blood sample	12 December 2000– 12	Maternal blood= normal range	Nil	Not applicable	There were positive correlations between mother's	Informed consent

	chromatography (HPLC).	was drawn from each mother and from the umbilical vein.	September 2002	38–150 (nmol/L); Cord Blood= normal range 30–150 (nmol/L)			vitamin D levels and infant's birth weight and between umbilical cord retinol concentrations and infant's birth weight.	
Narchi et al., 2010	Radio-immunoassay kit	Blood	September and November 2007	Vitamin D sufficiency was defined by a level >20 ng/ml, insufficiency when between 10 and 20 ng/ml, and deficiency when <10 ng/ml.	Adequate status: 30.2 (8.7) ng/ml (SD); Inadequate status: 11.6 (4.4) ng/ml (SD) at First Antenatal Visit		Blood was collected at the first antenatal visit, at 3 days after birth and at 6 months after delivery. The women were seen at a median of 3 days and 24 weeks after delivery.	Written consent
Dawodu and Nath et al., 2011	High- performance liquid chromatography	Materna l serum and cord blood	Not mentioned	Not mentioned	Nil	Not applicable	The findings of this study confirm our hypothesis that the vitamin D stores of prematurely born Arab infants will be low because of the high prevalence of maternal vitamin D deficiency.	Informed consent
Dawodu et al., 2011	High performance liquid chromatography	Venous Blood	September to November 2001 (summer)	vitamin D deficiency= Serum 25(OH)D < 50 nmol/L (20 ng/mL), moderate	Nil	Not applicable	Despite a significant post-intervention increase in serum 25(OH)D concentrations, all the women still had subclinical vitamin	Consented to participate in the study.

				vitaminD deficiency= < 25 nmol/L			deficiency [serum 25(OH)Dlevels < 50 nmol/L] because of their very low baseline vitamin D status	
H Narchi, et al., 2011	Radio-immunoassay (RIA kit, Immuno Diagnostic Systems Ltd, UK)	Blood	September and November 2007	Vitamin D sufficiency 50 > nmol/L, insufficiency as 25–50 nmol/L and deficiency as < 25 nmol/L	Mean (SD) serum vitamin D level Maternal 35.5 nmol/L (24.7); Infants *at birth 44.7 (29.7) nmol/L	Mean (SD) serum vitamin D level Maternal 14.2 ng/mL (9.9); Infants *at birth 17.9 (11.9) ng/ml	Serum 25-OH-vitamin-D levels were adequate in 8 infants (30%, CI 14–50%), insufficient in 13 (48%, CI 28–60%) and deficient in six (22%, CI 8.5–42%).	Not mentioned
Fatme Al Anouti, et al., 2011	Waters HPLC 2695 separation module with UV detection using Chromsystems kits (Chromsystems Instruments & Chemicals GmbH, Heimburgstrasse, Munich, Germany) by using a modified high-performance liquid chromatography (HPLC) method.	Blood	Winter & summer	Deficiency: <25 nmol/L, sufficiency: 50–200 nmol/L and toxicity: >200 nmol/L.	Nil	Not applicable	The mean serum 25(OH)D concentration for females tested in winter was 31.3 ± 12.3 nmol/L while in the summer, it was 20.9 ± 14.9 nmol/L. This difference was statistically significant, suggesting that seasonal variation plays an important role in vitamin D status in the United Arab Emirates.	Written informed consent was obtained from all participants before study enrollment.
Rajah et al., 2012	Diasorin RIA at Biomnis Laboratories	Blood	From 2005 to 2008	deficiency < 25 nmol/l, insufficiency, 25–50 nmol/l, sufficiency > 50 nmol/l.	0–0.9 y= 68.8 (46.9), 1–1.9 y= 77.4 (33.4), 2–7.9 y= 53.1 (29.3), 8 -14 y= 35.3 (23.7) nmol/L	0–0.9 y= 27.6 (18.8), 1–1.9 y= 31 (13.4), 2–7.9 y= 21.3 (11.7), 8 -14 y= 14.1 (9.5) ng/ml	17.7% had vitamin D deficiency	Permission from parents

Muhairi et al., 2013	Radioimmunoassay (DiaSorin, Stillwater, MN)	Venous blood	March and April of 2010	Vitamin D deficient as having serum 25OHD level ≤15 ng/mL [≤37.5 nmol/L] and vitamin D insufficient as 25OHD level ≤20 ng/mL [≤50 nmol/L], respectively.	Nil	Not applicable	Overall 65.1% of study participants were either vitamin D deficient or insufficient. The prevalence of vitamin D deficiency varied between boys (10%) and girls (28%).	Provided informed consent
Al-Anouti et al., 2013	Diasorin (LIAISON) and high performance liquid chromatography (HPLC)	Blood	October 2010 and May 2011	sufficient= serum 25(OH)D>75 nmol/L,	Nil	Not applicable	sufficient =2.1%, severely deficient= 63.2%, deficient=29.1%, insufficient=5.7%	Written informed consent was obtained from all participants before study enrollment
Narchi, et al., 2015	Electro-chemiluminescence assay 'ECLIA' (ROCHE Cobas e411 analyser).	Blood	1 September 2011 to 31 May 2012	severe deficiency <27.5 nmol/L; deficiency 27.5–50.0 nmol/L; insufficiency 50–75 nmol/L; and sufficiency ≥ 75 nmol/L	vitamin D-sufficient= 1 (0.3), Vitamin D insufficiency (serum levels 50–75 nmol/L)= 3 (1), Deficiency= 58 (19.8), and severe deficiency= 231 (78.8) n(%)	vitamin D-sufficient= 0.4 (0.1), Vitamin D insufficiency (serum levels 20–30)= 1.2 (0.4), Deficiency= 23.2 (7.9), and severe deficiency= 92.5 (31.6) ng/ml	vitamin D-sufficient= 1 (0.3), Vitamin D insufficiency (serum levels 50–75 nmol/L)= 3 (1), Deficiency= 58 (19.8), and severe deficiency= 231 (78.8) n(%)	Signed informed consent was obtained from all the participants and their guardians.
Yammine et al., 2016	Immunofluorescence (Liaison XL)	Blood	February 2012 and January 2014.	deficiency 25(OH) D < 30 ng/mL; insufficiency 30–50	Males [N=2418 (30.5)] - 20.6 (11.3) ng/ml; Females [N=5506	Conversion not needed	vitamin D deficiency was significantly associated with the 17−31 years age group, males and	Not mentioned

				ng/mL; and sufficiency > 50 ng/mL.	(69.5)] - 19.8 (11.29) ng/ml; Locals [N=1137 (14.3)] 19.1 (11.1) ng/ml; Non-locals [N=6787 (85.7)] 20.07 (11.3)		Emirati nationality. < 30 ng/ml N=6767 (85.4); 30–50 ng/ml N=996 (12.5); > 50 ng/ml N=161 (2.1)	
Al Anouti, et al., 2017	DiaSorin (LIAISON ®, Saluggia, Italy)	Blood	November 2014 to November 2015	VTD insufficiency ≤50nmol/l; VTD sufficiency >50nmol/l	insufficient (≤ 50nmol/L)= 26.23 ± 0.97; sufficient (> 50nmol/L)= 71.92 ± 4.43 nmol/l	insufficient (≤ 20 ng/mL)= 10.5 ± 0.4; sufficient (> 20 ng/mL)= 28.8 ± 1.8 ng/mL	Data strongly suggest that genetic variants relevant to VTD metabolism could play an important role in defining VTD status among the young adult Emirati population.	The subjects were informed about the study and signed an informed written consent prior to participation.
Bani-issa et al., 2017	Not mentioned	Not mentioned	March to August of 2015	Cut-off value for vitamin D deficiency is serum (25(OH) D) of ≤31 nmol/L.	Nil	Not applicable	Only 26% of the total subjects had sufficient vitamin D levels whereas 74% had vitamin D deficiency (vitamin D serum level ≤ 30 nmol/L). Emirati participants had higher odds of having VDD compared to non-Emiratis.	Signed informed consent
Inman et al., 2017	Beckman Coulter Access 2 Immunoassay System (Beckman Coulter, USA)	Blood	January 2015 to April 2015	Not mentioned	Nil	Not applicable	Prospective serial analysis of this axis may identify predictive biomarkers of obesity and cardiometabolic	All participants in the pilot study read and understood the

							dysfunction in the UAEHFS.	information brochure and signed informed consent prior to recruitment.
A Hasan et al., 2017	Electrochemiluminescence immunoassay using a Cobas autoanalyzer (Roche Diagnostics, Germany)	Blood	Not mentioned	Not mentioned	Nil	Not applicable	About 15% had MetS with serum vitamin D levels of 25.5(18.2) nmol/L. VDR genotyping yielded: FokI: 57.1% FF and 38.9% Ff, BsmI:29.8% bb and 51.5% Bb, while TaqI showed 39.4% TT and 43.4% Tt.	Written informed consents
Thomas et al., 2017	DiaSorin's LIAI- SON analyzer and a technique known as high performance liquid chromatography (HPLC)	Blood	fall term, October to January	VTD deficient individuals (<20 nmol/L)	Not mentioned	Not applicable	Based on conservative guidelines for assessing VTD deficiency (Grant 2009; Sabetta et al. 2005) the rate deficiency rate (< 20 nmol/L) in the present study was 53.5%.	written informed consent
Nimri et al., 2018	Radioimmunoassay kit (BioSource, Brussels, Belgium)	Not mentioned	January-May	A normal serum level of vitamin D was defined as a 25(OH)D concentration >20 ng/mL, and vitamin D insufficiency as serum	Nil	Not applicable	Out of the 480 students, 180 (37.5%) were wearing hijab for religious or cultural reasons. The 180 students had subnormal serum 25 (OH)D levels (<20 ng/ml) . This is the first study to report	Students were informed that participation is voluntary, all responses will be anonymous and would

				25(OH)D < 20 ng/ml (50 nmol/L), while <10 ng/mL was considered as severe deficiency			on vitamin D status, and risk factors in female college students in Sharjah, UAE. Serum 25(OH)D deficiencies were recorded for 47.92% of the students.	remain confidential, and that participation in the survey implied consent.
Abdulle et al., 2019	Beckman Coulter Access 2 Immunoassay System	Blood specimens	Not mentioned	Not mentioned	Controls - 29.3 ± 13.4 ng/ml	Conversion not needed	The finding that levels of fasting plasma insulin were significantly higher in the control vs. T2D case subjects (133.6 ± 149.9 vs. 107.5 ± 93.3) was unexpected.	Signed an informed consent
Al Zarooni et al., 2019	Not mentioned	Not mentioned	October 2011 and November 2012	Deficient (< 50 nmol/L), insufficient (50–74 nmol/L) and normal (≥ 75 nmol/L).	Nil	Not applicable	Vast majority (72%) were vitamin D deficient, 10% were vitamin D insufficient, and only 4.1% had normal vitamin D levels	Written informed consent was obtained
Sharif-Askari et al., 2020	chemiluminescent immunoassay (DiaSorin, LIAISON 25®Vitamin D TOTAL Assay	Blood	Not mentioned	Deficient (<20 ng/mL), sufficient (20–50 ng/mL), and high serum 25(OH)D concentration (>50 ng/mL)	median (IQR) (Insulin Sensitive= 31.20 (21.4) ng/ml	Conversion not needed	The proportion of 25(OH)D deficiency was higher for insulin-resistant individuals as well (22% vs. 18%). Univariate Analysis and Multivariate Analysis were used in statistical analysis.	Written informed consent

Al-Amad et al., 2020	Electrochemiluminescence immunoassay	venous blood	October 2017 and March 2019	Not mentioned	Nil	Not applicable	Normal Vitamin D n= 22 (44.9); Low vitamin D n= 30 (54.5)	Informed consent was obtained from all individuals participants included in the study
Saeed et al., 2021	Not mentioned	Blood	23\February to 2\March 2020	Deficiency (<10 ng/dl), Insufficiency (10–29 ng/dl), Sufficiency (30–100 ng/dl), and potential intoxication (>150 ng/dl).	Vitamin D deficiency - 48 (16.70%); Vitamin D insufficiency - 196 (68.30%); Normal - 43 (15%) ng/dl	Vitamin D deficiency - 0.48 (16.70%); Vitamin D insufficiency - 1.96 (68.30%); Normal - 0.43 (15%) ng/ml	VDD and VDI were highly prevalent among 85% of the students. The mean BMI was (24.32±6.3) kg/m. The results showed a significant positive correlation between VDI and VDD with gender and students who were previously diagnosed with VDD; however, they were not statistically significant with other factors. The tonsillitis incidents were significantly associated with VDD, while no significant correlation with other incidences of RTIs was found.	All the participants signed a consent form.
Gariballa et al., 2022	Fully automated COBAS e411 analyzer that uses a patented Electro Chemiluminescence (ECL) technology for	Blood	Not mentioned	Deficiency (<20 ng/mL) vs. insufficiency (20– 32 ng/mL) or	Nil	Not applicable	25(OH)D did show however significant association with age, gender and type 2 diabetes and HbA1c.	Written consent obtained from all patients

	immunoassay analysis) from ROCHE diagnostics, Manheim.			optimal (>32 ng/mL)].				recruited to this study.
Al Zarooni et al., 2022	Not mentioned	Not mention ed	October 2011 and November 2012	Normal (≥ 75 nmol/L); Insufficiency (50–74 nmol/L); Deficiency (< 50 nmol/L)	Nil	Not applicable	Normal (≥ 75 nmol/L)= 5.1%, Insufficiency (50–74 nmol/L=15.3%, Deficiency (< 50 nmol/L)=79.6%	Written informed consent was obtained
AlAnouti et al., 2022	Radioimmunoassay (DiaSorin, Stillwater, Minnesota, MN, USA)	Venous blood	Not Mentioned	Vitamin D deficiency= 25-hydroxyvitamin D concentrations ≤20 ng/mL (50 nmol/L).	Nil	Not applicable	25(OH)D concentrations= 30 ± 11 in the Filipinas, 14 ± 10 in Arabs, and 15 ± 9 in South Asians.	study participants provided written informed consent.
AlAnouti et al., 2022	Chemiluminescence Unicel DXI immunoassay system (Beckman-coulter, USA)	Blood	Not mentioend	Vitamin D deficient <20 ng/ml, insufficiency (20–30 ng/ml)	Females - 18.7 (13.3, 27.2); Males - 19.8 (16.5, 25.3) ng/ml	Conversion not needed	vitamin D insufficiency=209 (52.4%), vitamin D deficiency=121 (30.3%), vitamin D sufficien= 69 (17.3%)	All participants read and understood the information leaflet and signed the consent form prior to recruitment
Majeed et al., 2023	Elecsys total II kit for Cobas platform (Roche Diagnostics, Indianapolis, Indiana)	Venous Blood	Not mentioned	25(OH)D levels as deficient (< 30 nmol/L), insufficient (30-50 nmol/L) or	NG controls= 34.9 (23.6, 50.5) (nmol/L)	Conversion not needed	25(OH)D deficiency was observed in 40.5% (n = 120) NG participants.	Informed consent

				sufficient (> 50 nmol/L)				
Alzohily et al., 2024	The Ultra-High-Performance Liquid Chromatography-Tandem Mass Spectrometry (UHPLC-MS/MS) method was developed and validated for the analysis of vitamin D metabolites in the serum.	Blood	Not mentioned	Vitamin D deficiency < 20 ng/ml, insufficiency between 21 and 29 ng/ml and optimal > 30 ng/ml)	Nil	Not applicable	The baseline and healthy subjects had comparable concentration of vitamin D2 and D3. However, healthy subjects had a higher concentration of 25OHD and its epimer compared to the baseline subjects.	Participants provided their consent
Jutell et al., 2024	Electrochemiluminescence binding assay using LIAISON	Blood	27th September until 31st December 2021	Severe deficiency <25nmol/L, moderate deficiency 25–49.9nmol/l, normal status ≥50nmol/l	Group I=17.5 ± 4.9 nmol/L; Group II= 37.3 ± 7.55 nmol/L; Group III=81.7 ± 23.73 nmol/L	Group I= 7± 2 ng/mL; Group II= 14.9 ± 3 ng/mL; Group III=32.7 ± 9.5 ng/mL	We observed a strong correlation across varying levels of maternal 25(OH)D, maintaining a consistent maternal/UCB transition despite the presence of common maternal comorbidities, also in caesarean section in the second stage of labor but with a lower probability.	signed the informed consent

Table S2. The individual scoring for the evaluated studies of the systematic review

Author	Defintion of controls D	Reported value	assurance of lab ı	Ethics committee	nple size calculatio	Total
Dawodu et al., 1998	0	1	0	1	0	2
Dawodu et al., 2001	1	1	0	0	0	2
Dawodu et al., 2003	0	0	0	1	0	1
Saadi et al., 2004	1	1	0	1	0	3
Saadi et al., 2006	0	1	0	1	0	2
Saadi et al., 2009	0	0	0	1	0	1
Amirlak et al., 2009	1	1	0	1	0	3
Narchi et al., 2010	1	1	0	1	1	4
Dawodu and Nath et al., 2011	0	1	0	1	0	2
Dawodu et al ., 2011	1	1	0	1	0	3
Narchi et al., 2011	0	0	0	1	0	1
Al Anouti et al., 2011	1	1	0	1	0	3
Rajah et al., 2012	1	1	0	1	0	3
Muhairi et al., 2013	1	1	1	1	0	4
Al-Anouti et al, 2013	0	1	0	1	0	2
Narchi et al., 2015	1	1	0	1	0	3
Yammine et al., 2016	1	1	0	1	0	3
Al Anouti et al., 2017	1	0	0	1	0	2
Bani-issa et al., 2017	1	0	0	1	0	2
Inman et al., 2017	1	1	0	1	0	3
A Hasan et al., 2017	0	1	0	1	0	2
Thomas et al., 2017	1	1	0	1	0	3
Nimri et al., 2018	1	1	0	1	0	3
Abdulle et al., 2019	1	1	0	1	0	3
Al Zarooni et al., 2019	1	0	0	1	0	2
Sharif-Askari et al., 2020	1	1	0	1	1	4
Al-Amad et al. 2020	1	1	1	1	0	4
Saeed et al ., 2021	1	1	0	1	0	3
Gariballa et al., 2022	1	1	0	1	0	3
Al Zarooni et al., 2022	1	1	0	1	0	3
Anouti et al., 2022	1	1	1	1	1	5
AlAnouti et al., 2022	0	1	0	1	0	2
Majeed et al., 2023	1	0	0	1	0	2
Alzohily et al., 2024	1	1	0	1	0	3
Jutell et al., 2024	1	0	0	1	0	2
